# A Redundant Configuration of Four Low-Cost GNSS-RTK Receivers for Reliable Estimation of Vehicular Position and Posture

**DOI:** 10.3390/s21175853

**Published:** 2021-08-30

**Authors:** Jesús Morales, Jorge L. Martínez, Alfonso J. García-Cerezo

**Affiliations:** Robotics and Mechatronic Lab, Andalucía Tech, Universidad de Málaga, 29071 Málaga, Spain; jlmartinez@uma.es (J.L.M.); ajgarcia@uma.es (A.J.G.-C.)

**Keywords:** vehicle localization, GNSS receivers, RTK corrections, sensor redundancy

## Abstract

This paper proposes a low-cost sensor system composed of four GNSS-RTK receivers to obtain accurate position and posture estimations for a vehicle in real-time. The four antennas of the receivers are placed so that every three-antennas combination is optimal to get the most precise 3D coordinates with respect to a global reference system. The redundancy provided by the fourth receiver allows to improve estimations even more and to maintain accuracy when one of the receivers fails. A mini computer with the Robotic Operating System is responsible for merging all the available measurements reliably. Successful experiments have been carried out with a ground rover on irregular terrain. Angular estimates similar to those of a high-performance IMU have been achieved in dynamic tests.

## 1. Introduction

Acquiring accurate and reliable three-dimensional (3D) coordinates for a vehicle is of great interest in monitoring its operation for advanced driver assistance systems and for autonomous navigation of mobile robots. Vehicle coordinates include three distances for position and three angles for posture with respect to a global reference system on Earth surface, that usually employs North-East-Down (NED) local axes [1].

A common possibility is the use of an Inertial Measurement Unit (IMU), which contain different sensors such as accelerometers, gyroscopes and magnetometers [2]. For proper operation, these units require calibration once installed on the vehicle and to take into account local magnetic field variations. Knowing the initial position of the vehicle, global 3D coordinates can be obtained with an IMU and odometry, but the estimation of the spatial trajectory tends to deteriorate since the measurements include small deviations that accumulate over time [3].

To avoid the growth of position uncertainty, a Global Navigation Satellite System (GNSS) receiver that make use of various global satellite constellations (North American GPS, Russian GLONASS, European Galileo and Chinese BeiDou) at once can be employed [4]. However, absolute GNSS measurements over the Earth’s surface are subject to various types of errors that degrade their accuracy to the order of meters.

GNSS errors can be significantly reduced by incorporating differential corrections provided by a Satellite Based Augmentation Systems (SBAS) or a Continuously Operating Reference Station (CORS) [5]. In this respect, one of the most effective techniques is Real Time Kinematics (RTK) that performs carrier-phase signal synchronization [6] by using the RTCM (Radio Technical Commission for Maritime Services) communication protocol. Thus, GNSS receivers can operate in two different modes: RTK-fixed and RTK-float to indicate when they achieve or not centimeter accuracy, respectively.

Besides, multiple GNSS receivers can be installed onboard to enhance vehicle positioning [5,7]. In addition, a GNSS compass with two antennas can be employed to obtain heading [8,9]. Moreover, by differencing over time the GNSS measurements taken in motion, speed estimations [10] as well as pitch and heading [11] can be deduced.

In field robotics, the combination of GPS and IMU sensors has been a popular option to estimate 3D global coordinates accurately [12]. A different strategy to achieve this objective was to mount the antennas of three high-cost GPS-RTK receivers on the roof of the vehicle [13].

With modern GNSS receivers, the GNSS-IMU sensor combination [14] and the synchronization of three low-cost GNSS-RTK devices [6,15] or three antennas in a single receiver [16] have also been employed in automobiles. Moreover, by tightly coupling three GNSS-RTK receivers and an IMU, accuracy can be improved even more [1,17,18].

This paper proposes a reliable sensor system that provides the position and posture of a vehicle by combining the measurements of four identical GNSS-RTK low-cost receivers. In this way, the main contributions are the following:

1. The best geometrical configuration for three and four antennas to minimize position and posture uncertainty of a vehicle is deduced.
2. A redundant setup with four antennas is analyzed, so when the precision of one receiver degrades, reliable 3D coordinates can be still calculated in real-time.
3. A decentralized node architecture using the Robot Operating System (ROS) that integrates all the available measurements from the receivers is presented.

Regarding the first point, although several antenna configurations have been deployed experimentally on cars, no previous work has performed a theoretical analysis to infer the best layout. This has not prevented two recent papers [6,16] from employing near-optimal configurations for their tests.

With respect to the second contribution, antenna redundancy was previously intended only to improve positioning precision [5,7], but in this paper it also serves to enhance attitude estimations for the vehicle and to tolerate faults on the GNSS receivers.

Regarding the third point, following a low-cost philosophy, it is employed an open-source software of common use in robotics with some already developed nodes in a mini computer instead of programming on specialized boards [6,15,16].

The rest of the paper is organized as follows. Antenna arrangements with three and four GNSS-RTK receivers are discussed in the next Section. Sensor hardware, ROS programming and the optimal calculation of 3D coordinates are described in Section 3. Then, experiments on irregular terrain with the robotic platform Argo XTR are presented in Section 4, including comparisons with measurements from a high-end IMU. Finally, conclusions, acknowledgements, and references complete the paper.

## 2. Spatial Configurations for the Antennas of the GNSS-RTK Receivers

Let $v_{i}$ be the actual position of an antenna with respect to a global NED coordinate system:

(1) $$v_{i}\left(t\right)=\left(\begin{array}{c} x_{i}\left(t\right)\\ y_{i}\left(t\right)\\ z_{i}\left(t\right) \end{array}\right)={\bar{v}}_{i}\left(t\right)+\Delta v_{i}\left(t\right)=\left(\begin{array}{c} {\bar{x}}_{i}\left(t\right)\\ {\bar{y}}_{i}\left(t\right)\\ {\bar{z}}_{i}\left(t\right) \end{array}\right)+\left(\begin{array}{c} \Delta x_{i}\left(t\right)\\ \Delta y_{i}\left(t\right)\\ \Delta z_{i}\left(t\right) \end{array}\right),$$

where ${\bar{v}}_{i}\left(t\right)$ is the measurement produced by the receiver i=1,2,3,4 at instant *t* and $\Delta v_{i}\left(t\right)$ is its associated error. The x,y,z coordinates correspond to north, east, and down displacements, respectively. The covariance matrix for $\Delta v_{i}$ is given by:   

(2) $$\begin{array}{cc} C_{i}=E\left[\Delta v_{i}\left(t\right)\;\Delta v_{i}^{T}\left(t\right)\right] & =\\ E\left[\left(\begin{array}{c} \Delta x_{i}\left(t\right)\\ \Delta y_{i}\left(t\right)\\ \Delta z_{i}\left(t\right) \end{array}\right)\left(\Delta x_{i}\left(t\right),\;\Delta y_{i}\left(t\right),\;\Delta z_{i}\left(t\right)\right)\right] & =\left(\begin{array}{ccc} \sigma_{x_{i}}^{2} & \sigma_{x_{i}y_{i}} & \sigma_{x_{i}z_{i}}\\ \sigma_{x_{i}y_{i}} & \sigma_{y_{i}}^{2} & \sigma_{y_{i}z_{i}}\\ \sigma_{x_{i}z_{i}} & \sigma_{y_{i}z_{i}} & \sigma_{z_{i}}^{2} \end{array}\right), \end{array}$$

where $\sigma_{x_{i}}^{2}=E\left[\Delta x_{i}^{2}\left(t\right)\right]$, $\sigma_{y_{i}}^{2}=E\left[\Delta y_{i}^{2}\left(t\right)\right]$ and $\sigma_{z_{i}}^{2}=E\left[\Delta z_{i}^{2}\left(t\right)\right]$ are the variances, whereas $\sigma_{x_{i}y_{i}}=E\left[\Delta x_{i}\left(t\right)\Delta y_{i}\left(t\right)\right]$, $\sigma_{x_{i}z_{i}}=E\left[\Delta x_{i}\left(t\right)\Delta z_{i}\left(t\right)\right]$ and $\sigma_{y_{i}z_{i}}=E\left[\Delta y_{i}\left(t\right)\Delta z_{i}\left(t\right)\right]$ are the covariances.

For this analysis, it is considered that:

- The mean errors of the receivers along time are null, i.e., $E[\Delta v_{i}\left(t\right)]=0\;\forall i$.
- The errors of different receivers are independent, i.e., $E[\Delta v_{i}\left(t\right)\Delta v_{j}^{T}\left(t\right)]=0$ for i≠j.
- All the receivers share the same covariance matrix, i.e., $C=C_{1}=C_{2}=C_{3}=C_{4}$.

### 2.1. Three Receivers Optimal Configuration

To obtain the optimal configuration for the triangle formed by three GNSS-RTK antennas, its associated position and posture uncertainty should be minimized. In this case, it is also assumed that:

- The distances $d_{a}$ and $d_{b}$ of the second and third antenna with respect to the first antenna, respectively, are constant values determined without uncertainty.
- The angle θ between the directions given by $d_{a}$ and $d_{b}$ is also known certainly on the plane that contains the three antennas.

The centroid of the triangle formed by the antennas:

(3) $$v_{0}\left(t\right)=\frac{v_{1}\left(t\right)+v_{2}\left(t\right)+v_{3}\left(t\right)}{3},$$

can be estimated from the measurements of the receivers as:

(4) $${\bar{v}}_{0}\left(t\right)=\frac{{\bar{v}}_{1}\left(t\right)+{\bar{v}}_{2}\left(t\right)+{\bar{v}}_{3}\left(t\right)}{3}\Rightarrow \Delta v_{0}\left(t\right)=\frac{\Delta v_{1}\left(t\right)+\Delta v_{2}\left(t\right)+\Delta v_{3}\left(t\right)}{3}.$$

The covariance matrix for $\Delta v_{0}$ is calculated as:   

(5) $$C_{0}=E\left[\Delta v_{0}\left(t\right)\Delta v_{0}^{T}\left(t\right)\right]=\frac{E\left[\Delta v_{1}\left(t\right)\Delta v_{1}^{T}\left(t\right)+\Delta v_{2}\left(t\right)\Delta v_{2}^{T}\left(t\right)+\Delta v_{3}\left(t\right)\Delta v_{3}^{T}\left(t\right)\right]}{9}=\frac{C}{3},$$

where the position uncertainty of the geometric center of the triangle is reduced by three with respect to each vertex.

Regarding the posture in space of the triangle, the direction cosines of the lines between two antennas are given by the unitary vectors:  

(6) $$\begin{array}{cc} \; & v_{a}\left(t\right)=\frac{v_{2}\left(t\right)-v_{1}\left(t\right)}{d_{a}}=\left(\begin{array}{c} x_{a}\left(t\right)\\ y_{a}\left(t\right)\\ z_{a}\left(t\right) \end{array}\right)\Rightarrow \end{array}$$

(7) $$\begin{array}{cc} & {\bar{v}}_{a}\left(t\right)=\frac{{\bar{v}}_{2}\left(t\right)-{\bar{v}}_{1}\left(t\right)}{d_{a}},\;\;\Delta v_{a}\left(t\right)=\frac{\Delta v_{2}\left(t\right)-\Delta v_{1}\left(t\right)}{d_{a}}, \end{array}$$

(8) $$\begin{array}{cc} \; & v_{b}\left(t\right)=\frac{v_{3}\left(t\right)-v_{1}\left(t\right)}{d_{b}}=\left(\begin{array}{c} x_{b}\left(t\right)\\ y_{b}\left(t\right)\\ z_{b}\left(t\right) \end{array}\right)\Rightarrow \end{array}$$

(9) $$\begin{array}{cc} & {\bar{v}}_{b}\left(t\right)=\frac{{\bar{v}}_{3}\left(t\right)-{\bar{v}}_{1}\left(t\right)}{d_{b}},\;\;\Delta v_{b}\left(t\right)=\frac{\Delta v_{3}\left(t\right)-\Delta v_{1}\left(t\right)}{d_{b}}, \end{array}$$

where the corresponding covariance matrices are:

(10) $$\begin{array}{cc} C_{a}= & E\left[\Delta v_{a}\left(t\right)\Delta v_{a}^{T}\left(t\right)\right]=\frac{E\left[\Delta v_{2}\left(t\right)\Delta v_{2}^{T}\left(t\right)\right]}{d_{a}^{2}}+\frac{E\left[\Delta v_{1}\left(t\right)\Delta v_{1}^{T}\left(t\right)\right]}{d_{a}^{2}}=\frac{2C}{d_{a}^{2}}, \end{array}$$

(11) $$\begin{array}{cc} C_{b}= & E\left[\Delta v_{b}\left(t\right)\Delta v_{b}^{T}\left(t\right)\right]=\frac{E\left[\Delta v_{3}\left(t\right)\Delta v_{3}^{T}\left(t\right)\right]}{d_{b}^{2}}+\frac{E\left[\Delta v_{1}\left(t\right)\Delta v_{1}^{T}\left(t\right)\right]}{d_{b}^{2}}=\frac{2C}{d_{b}^{2}}, \end{array}$$

(12) $$\begin{array}{cc} C_{ab}= & E\left[\Delta v_{a}\left(t\right)\Delta v_{b}^{T}\left(t\right)\right]=C_{ba}=E\left[\Delta v_{b}\left(t\right)\Delta v_{a}^{T}\left(t\right)\right]=\frac{E\left[\Delta v_{1}\left(t\right)\Delta v_{1}^{T}\left(t\right)\right]}{d_{a}\;d_{b}}=\frac{C}{d_{a}\;d_{b}}. \end{array}$$

Thus, the spatial uncertainty of the direction cosines can be reduced by separating the antennas as much as possible. But $d_{a}$ and $d_{b}$ are inherently limited by the available space on the roof of the vehicle. Furthermore, to balance posture uncertainty in both directions, these distances should be selected equal: $d=d_{a}=d_{b}$, so that $C_{a}=C_{b}=2C/d^{2}$ and $C_{ab}=C_{ba}=C/d^{2}$.

The direction cosine of the normal vector $v_{c}$ to the plane defined by the three antennas is given by the unitary vector from the cross product of $v_{a}$ and $v_{b}$:

(13) $$\begin{array}{cc} v_{c}\left(t\right)= & \frac{v_{a}\left(t\right)\times v_{b}\left(t\right)}{\sin(\theta )}=\frac{1}{\sin(\theta )}\left(\begin{array}{c} y_{a}\left(t\right)z_{b}\left(t\right)-y_{b}\left(t\right)z_{a}\left(t\right)\\ x_{b}\left(t\right)z_{a}\left(t\right)-x_{a}\left(t\right)z_{b}\left(t\right)\\ x_{a}\left(t\right)y_{b}\left(t\right)-x_{b}\left(t\right)y_{a}\left(t\right) \end{array}\right)\Rightarrow \end{array}$$

(14) $$\begin{array}{cc} {\bar{v}}_{c}\left(t\right)= & \frac{1}{\sin(\theta )}\left(\begin{array}{c} {\bar{y}}_{a}\left(t\right){\bar{z}}_{b}\left(t\right)-{\bar{y}}_{b}\left(t\right){\bar{z}}_{a}\left(t\right)\\ {\bar{x}}_{b}\left(t\right){\bar{z}}_{a}\left(t\right)-{\bar{x}}_{a}\left(t\right){\bar{z}}_{b}\left(t\right)\\ {\bar{x}}_{a}\left(t\right){\bar{y}}_{b}\left(t\right)-{\bar{x}}_{b}\left(t\right){\bar{y}}_{a}\left(t\right) \end{array}\right), \end{array}$$

as long as the three antennas are not aligned to avoid sin(θ)=0, where $v_{c}$ will be indeterminate.

By using Taylor series expansion [19], $v_{c}$ can be approximated by:

(15) $$v_{c}\left(t\right)\approx {\bar{v}}_{c}\left(t\right)+\frac{J(t)}{\sin(\theta )}\left(\begin{array}{c} \Delta v_{a}\left(t\right)\\ \Delta v_{b}\left(t\right) \end{array}\right)\Rightarrow \Delta v_{c}\left(t\right)\approx \frac{J(t)}{\sin(\theta )}\left(\begin{array}{c} \Delta v_{a}\left(t\right)\\ \Delta v_{b}\left(t\right) \end{array}\right),$$

where *J* is the time-dependent Jacobian matrix:

(16) $$J\left(t\right)=\left(\begin{array}{cccccc} 0 & {\bar{z}}_{b}\left(t\right) & -{\bar{y}}_{b}\left(t\right) & 0 & -{\bar{z}}_{a}\left(t\right) & {\bar{y}}_{a}\left(t\right)\\ -{\bar{z}}_{b}\left(t\right) & 0 & {\bar{x}}_{b}\left(t\right) & {\bar{z}}_{a}\left(t\right) & 0 & -{\bar{x}}_{a}\left(t\right)\\ {\bar{y}}_{b}\left(t\right) & -{\bar{x}}_{b}\left(t\right) & 0 & -{\bar{y}}_{a}\left(t\right) & {\bar{x}}_{a}\left(t\right) & 0 \end{array}\right).$$

The error $\Delta v_{c}$ can be minimized regardless of vehicle posture by choosing $\theta =\pm 90{}^{\circ }$. In this case, the covariance matrix for $\Delta v_{c}$ can be approximated by:

(17) $$C_{c}\left(t\right)=E\left[\Delta v_{c}\left(t\right)\Delta v_{c}^{T}\left(t\right)\right]\approx J\left(t\right)\left(\begin{array}{cc} C_{a} & C_{ab}\\ C_{ba} & C_{b} \end{array}\right)J^{T}\left(t\right)=\frac{J(t)}{d^{2}}\left(\begin{array}{cc} 2C & C\\ C & 2C \end{array}\right)J^{T}\left(t\right).$$

Finally, the rotation matrix is obtained from the direction cosines as $R=\left({\bar{v}}_{a},\;{\bar{v}}_{b},\;{\bar{v}}_{c}\right)$. Roll, pitch and yaw angles can be deduced from *R* and represent rotations with respect to $v_{a}$, $v_{b}$ and $v_{c}$ axis, respectively.

**Figure 1:**
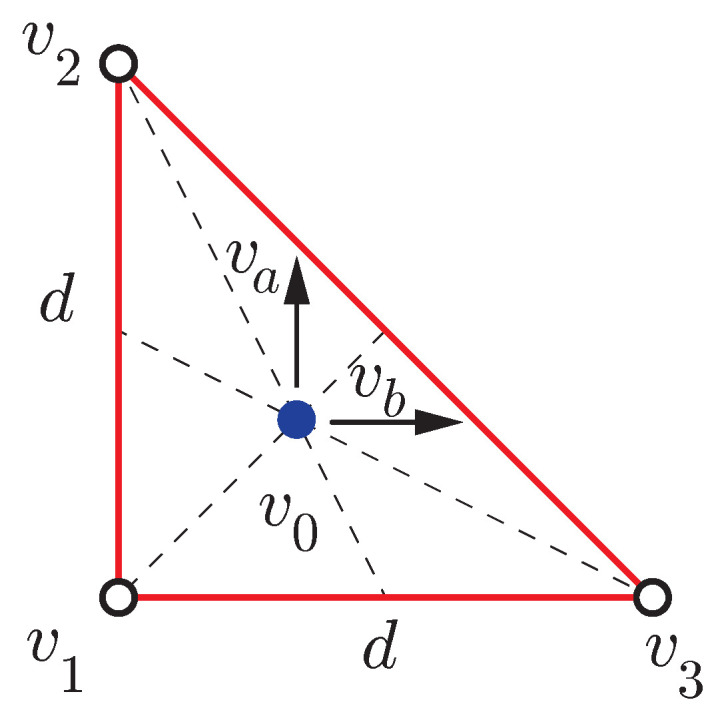
Optimal antenna configuration with three receivers.

To summarize, the best configuration to minimize position and posture uncertainty with three GNSS-RTK antennas is to form a right-angled triangle with two identical sides of the maximum possible length *d* (see Figure 1).

### 2.2. Four Receivers Optimal Layout

**Figure 2:**
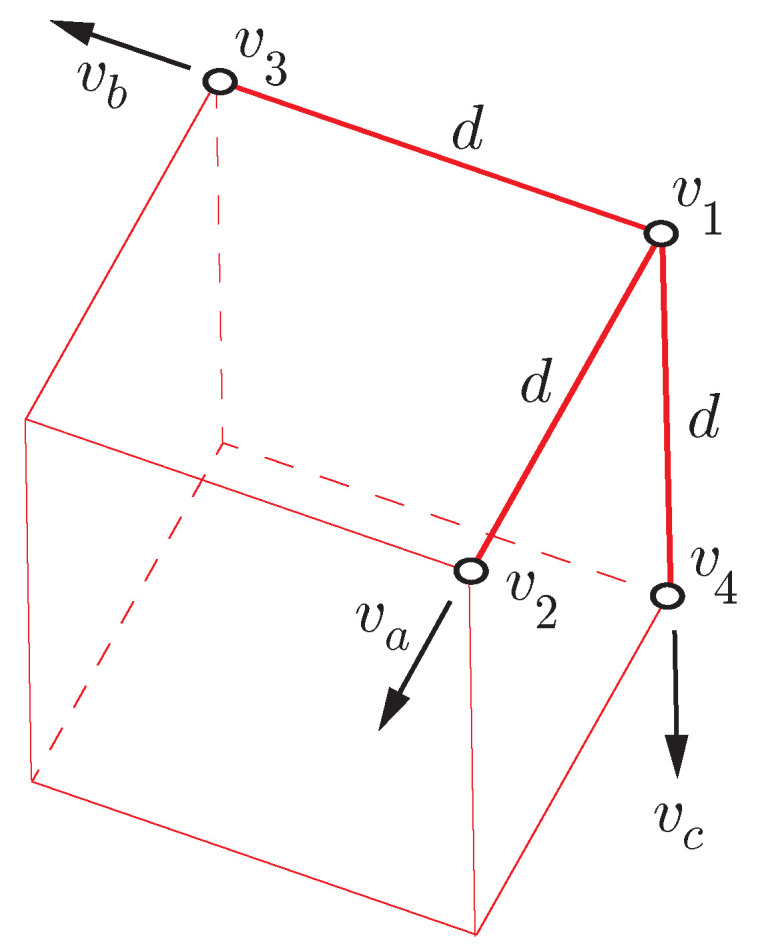
Optimal antenna configuration with four receivers.

The optimum for four receivers would consist of placing the fourth antenna orthogonal to the plane defined by the remaining three at a distance *d* of the first antenna, where its centroid:

(18) $$v_{0}\left(t\right)=\frac{v_{1}\left(t\right)+v_{2}\left(t\right)+v_{3}\left(t\right)+v_{4}\left(t\right)}{4},$$

does not coincide with the geometric center of the underlying cube (see Figure 2).

Assuming perfect placement of the fourth receiver with respect to the triangle:

(19) $$v_{c}\left(t\right)=\frac{v_{4}\left(t\right)-v_{1}\left(t\right)}{d},$$

which can be calculated directly from measurements as:

(20) $${\bar{v}}_{c}\left(t\right)=\frac{1}{d}\left(\begin{array}{c} {\bar{x}}_{4}\left(t\right)-{\bar{x}}_{1}\left(t\right)\\ {\bar{y}}_{4}\left(t\right)-{\bar{y}}_{1}\left(t\right)\\ {\bar{z}}_{4}\left(t\right)-{\bar{z}}_{1}\left(t\right) \end{array}\right)\Rightarrow \Delta v_{c}=\frac{\Delta v_{4}\left(t\right)-\Delta v_{1}\left(t\right)}{d},$$

and can be merged with the estimation (14) to decrease posture uncertainty even more. Furthermore, this reduction can also be applied to ${\bar{v}}_{a}$ (7) and ${\bar{v}}_{b}$ (9) with their corresponding normal vectors formed by their respective ortogonal planes: ${\bar{v}}_{a}\left(t\right)={\bar{v}}_{b}\left(t\right)\times {\bar{v}}_{c}\left(t\right)$ and ${\bar{v}}_{b}\left(t\right)={\bar{v}}_{c}\left(t\right)\times {\bar{v}}_{a}\left(t\right)$, respectively.

### 2.3. Four Receivers Redundant Configuration

**Figure 3:**
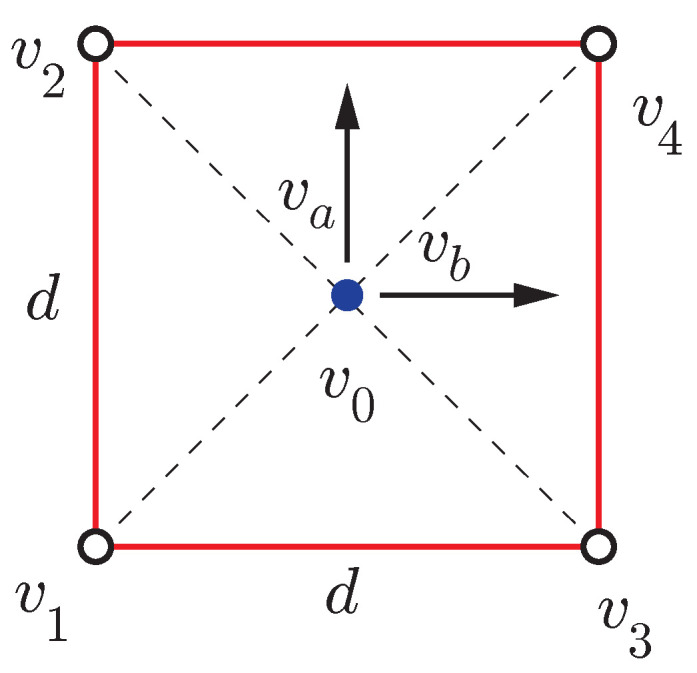
Redundant antenna configuration with four receivers.

**Figure 4:**
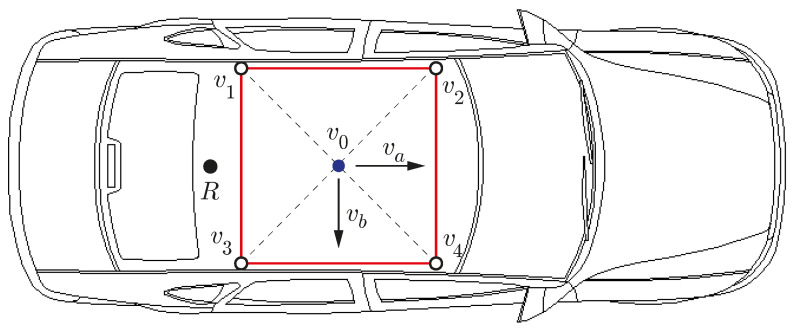
Placement of the antennas on the roof of a car.

In this paper, an additional fourth receiver is added to the optimal three-receivers configuration to form a square of side *d* on a planar surface (see Figure 3). This is a redundant arrangement that is easier to mount on the roof of an automobile (see Figure 4) than the optimal one of Figure 2. The local coordinate system has its origin in $v_{0}$ with axes $v_{a}$, $v_{b}$ and $v_{c}$ as defined by (7), (9) and (13), respectively.

In Figure 4, the longitudinal and transverse axes of the car coincide with $v_{a}$ and $v_{b}$, respectively. The vertical axis $v_{c}$ is not displayed but it would be pointing downwards. This figure also shows the position of a radio antenna, denoted by the letter R, to receive RTK corrections from a CORS.

The proposed redundancy is useful in two different ways. Firstly, position and posture uncertainty can be reduced further than with three receivers. Secondly, if the precision of one of the receivers deteriorates, the rest of receivers can still provide a reliable position and posture estimation for the vehicle.

Regarding the first advantage, when all the measurements are available, its centroid $v_{0}$ (18) can be estimated as:  

(21) $$\begin{array}{cc} \; & {\bar{v}}_{0}\left(t\right)=\frac{{\bar{v}}_{1}\left(t\right)+{\bar{v}}_{2}\left(t\right)+{\bar{v}}_{3}\left(t\right)+{\bar{v}}_{4}\left(t\right)}{4}\Rightarrow \end{array}$$

(22) $$\begin{array}{c} \Delta v_{0}\left(t\right)=\frac{\Delta v_{1}\left(t\right)+\Delta v_{2}\left(t\right)+\Delta v_{3}\left(t\right)+\Delta v_{4}\left(t\right)}{4}\Rightarrow C_{0}=\frac{C}{4}, \end{array}$$

whose covariance matrix is divided by four, instead of by three (5). Furthermore, the direction cosines $v_{a}$ and $v_{b}$ can be estimated by using one side of the square and its opposite:

(23) $$\begin{array}{cc} \; & {\bar{v}}_{a}\left(t\right)=\frac{{\bar{v}}_{2}\left(t\right)-{\bar{v}}_{1}\left(t\right)+{\bar{v}}_{4}\left(t\right)-{\bar{v}}_{3}\left(t\right)}{2d}\Rightarrow \end{array}$$

(24) $$\begin{array}{cc} & \Delta v_{a}\left(t\right)=\frac{\Delta v_{2}\left(t\right)-\Delta v_{1}\left(t\right)+\Delta v_{4}\left(t\right)-\Delta v_{3}\left(t\right)}{2d}\Rightarrow C_{a}=\frac{C}{d^{2}}, \end{array}$$

(25) $$\begin{array}{cc} \; & {\bar{v}}_{b}\left(t\right)=\frac{{\bar{v}}_{3}\left(t\right)-{\bar{v}}_{1}\left(t\right)+{\bar{v}}_{4}\left(t\right)-{\bar{v}}_{2}\left(t\right)}{2d}\Rightarrow \end{array}$$

(26) $$\begin{array}{cc} & \Delta v_{b}\left(t\right)=\frac{\Delta v_{3}\left(t\right)-\Delta v_{1}\left(t\right)+\Delta v_{4}\left(t\right)-\Delta v_{2}\left(t\right)}{2d}\Rightarrow C_{b}=\frac{C}{d^{2}}, \end{array}$$

that represents half uncertainty of (10) and (11). This reduction directly benefits to the covariance matrix $C_{c}$ (17) of the direction cosine $v_{c}$:

(27) $$\Delta v_{c}\left(t\right)\approx J\left(t\right)\left(\begin{array}{c} \Delta v_{a}\left(t\right)\\ \Delta v_{b}\left(t\right) \end{array}\right)\Rightarrow C_{c}\left(t\right)\approx \frac{J(t)}{d^{2}}\left(\begin{array}{cc} C & C\\ C & C \end{array}\right)J^{T}\left(t\right),$$

where $C_{ab}=C_{ba}=C/d^{2}$.

The second advantage comes from the fact that with a three-antenna configuration, there is no possibility to obtain the complete set of six coordinates for the vehicle when one of the receivers fails. However, the proposed sensor system can keep working with the remaining three receivers. In this case, to obtain the center of the square, instead of the triangle centroid (4), only two measurements from opposite vertices can be employed:

υ¯0(t)={(28)υ¯2(t)+υ¯3(t)2,when the first or fourth receiver fails,(29)υ¯1(t)+υ4(t)2,when the second or third receiver fails,

which implies that $C_{0}=C/2$, i.e., twice position uncertainty with respect to four available measurements (22).

## 3. Sensor System

**Figure 5:**
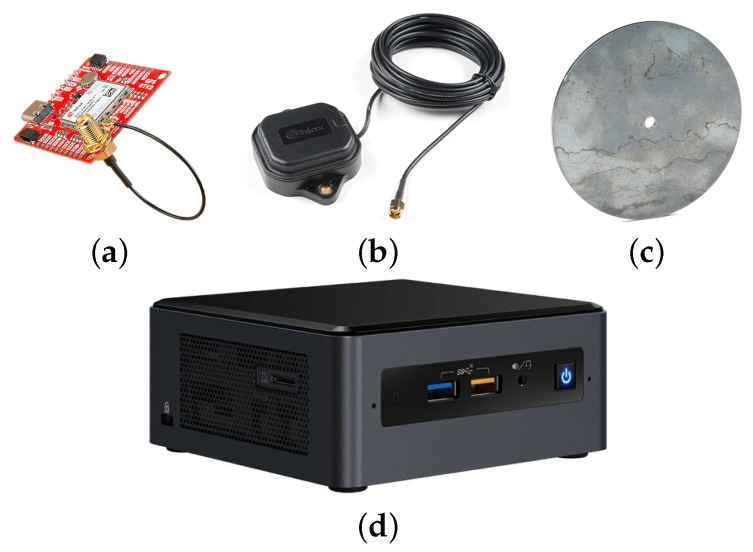
Hardware components of the sensor system: a GNSS-RTK board (**a**), an antenna (**b**), a ground plate (**c**) and the mini computer (**d**).

In addition to the four low-cost GNSS receivers and their corresponding antennas, the sensor system includes a mini computer to obtain the 3D position and posture of the vehicle (see Figure 5).

The chosen GNSS-RTK receiver is the SparkFun GPS-RTK2 (https://www.sparkfun.com/products/15136, accessed on 28 July 2021) board, which is based on the compact ZED-F9P module from U-blox. The receiver does not only provide geodetic coordinates (longitude, latitude and height), but also ECEF (Cartesian coordinates with respect to Earth center) and NED coordinates with respect to a nearby CORS to obtain centimeter accuracy with an output rate of 8 Hz.

Each receiver is connected to a multi-band antenna ANN-MB-00 (https://www.sparkfun.com/products/15192, accessed on 28 July 2021) from U-blox. To avoid multi-path problems, the magnetic base of each antenna is mounted on a steel ground plate.

The mini computer is an Intel NUC8i7BEH (https://www.intel.es/content/www/es/es/products/sku/126140/intel-nuc-kit-nuc8i7beh/specifications.html, accessed on 28 July 2021) kit with an Intel Core i7-8559U processor (8M Cache, 4 cores, 2.70 GHz). It has installed the open-source framework ROS [20] on the Linux-based operating system Ubuntu.

The computer communicates with each receiver through different Universal Serial Bus (USB) ports. The Internet connection of the computer is used to get differential correction data via the standard protocol NTRIP (Networked Transport of RTCM via Internet Protocol) through the Andalusian public positioning network [21].

### ROS Programming

**Figure 6:**
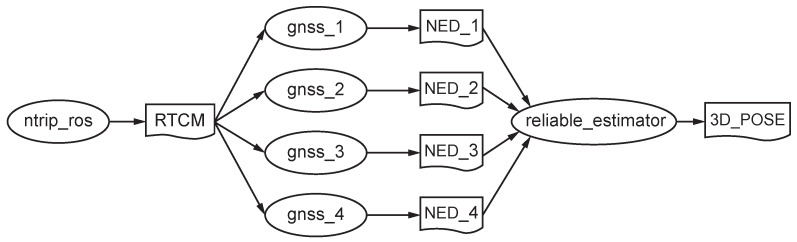
ROS computation graph with nodes (ellipses) and topics.

The developed ROS software consists of a number of independent nodes, each of which communicates with others using topics under a publish/subscribe messaging model (see Figure 6).

The ntrip_ros (https://github.com/ros-agriculture/ntrip_ros, accessed on 28 July 2021) node connects to a nearby CORS to get RTCM streams through internet and to publish them into the topic RTCM. Each receiver *i* has associated a driver node (https://github.com/KumarRobotics/ublox, accessed on 28 July 2021) named gnss_i that is subscribed to this topic to receive differential corrections via callbacks. These nodes publish NED coordinates on its own topic NED_i along with a time stamp and three accuracy estimates (each one ≥10 mm).

Then, the reliable_estimator node receives all the messages from the four NED_i topics and computes the six 3D coordinates with three or four synchronized measurements. Finally, it publishes the current pose into the 3D_POSE topic, making this data available for any navigation node on the ROS system.

All the receivers weight the same to produce vehicular position and attitude in real-time. When the accuracy of one receiver degrades, it is completely discarded from computations.

For calculating the global position of the centroid ${\bar{v}}_{0}$ of the square (21), (28) or (29) are employed depending on the number of valid GNSS-RTK measurements. For posture computation, the closed-form method by Horn [22] is applied with a scale factor of 1. Instead of quaternions, an orthonormal rotation matrix *R* [23] is obtained that minimizes the following cost function:

(30) $$F\left(t\right)=\sum_{\forall i}{∥\left(v_{i}^{*}-v_{0}^{*}\right)-R\left(t\right)\left({\bar{v}}_{i}\left(t\right)-{\bar{v}}_{0}\left(t\right)\right)∥}^{2},$$

where $v_{i}^{*}$ and $v_{0}^{*}$ are the relative positions of the antenna *i* and the centroid of the square with respect to the local reference system, respectively. This represents a least squares problem that can be solved with three or four valid measurements. Lastly, the roll, pitch and yaw angles with respect to the global NED axes are extracted from the resulting rotation matrix [17].

## 4. Experiments with the Rover Argo XTR

**Figure 7:**
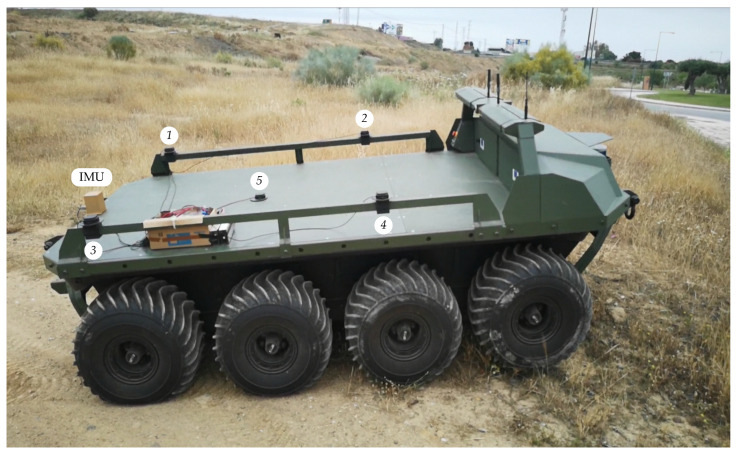
The rover Argo XTR with the sensor system mounted on the rear deck. GNSS antennas are numbered from 1 to 5.

The rover Argo XTR is a battery-powered unmanned land vehicle that allows extreme mobility with a low center of gravity and amphibious capability (see Figure 7). It features skid-steer traction with eight low-pressure 24-inch tires, a top speed of 16 km h^−1^ and zero turning radius. The robotic rover can be controlled via a follow-me system with a 2D laser scanner or via remote teleoperation with a joystick and a line-of-sight wireless link.

The proposed sensor has been mounted on the rear deck of the vehicle. The four antennas are tied to the side rails forming a square of d=1.35m on the side (see Figure 7). For comparison purposes, a fifth antenna has been installed at the center of the square together with the high-end AHRS400CC-100 MEMS IMU from Crossbow with an output rate of 60 Hz [24].

The Málaga broadcast station located 4.8 km away is employed to get differential corrections RTCM 3.1 through 4G internet connection and it is considered the global NED reference system in the following experiments.

### 4.1. Calibration Test

This test was carried out by recording the RTK-fixed measurements of the five GNSS receivers during three hours with the rover stopped on an almost horizontal parking lot. This experiment serves to characterize the covariance matriz *C* for the positioning errors. To this end, the mean NED coordinates are calculated for each receiver and the difference of each measurement with respect to its mean value is considered as an error. Then, by using (2):

(31) $$C=10^{-3}\left(\begin{array}{ccc} 0.0434 & -0.0025 & -0.0061\\ -0.0025 & 0.0694 & -0.0083\\ -0.0061 & -0.0083 & 0.4255 \end{array}\right)m^{2},$$

where it can be observed almost null covariances and a standard deviation in the *z* coordinate ($\sigma_{z}=21\;mm$) much greater than in the *x* ($\sigma_{x}=7\;mm$) and in the *y* ($\sigma_{y}=8\;mm$) coordinates.

Moreover, the relative location of each antenna $v_{i}^{*}$ can also be accurately estimated with the computed mean values (see Table 1). It can be observed small positioning errors on the square with the fifth antenna centered and 166 mm below the rest of antennas. Table 1 also includes the local position of the centroids of the square $v_{0}^{*}$ for (30) when using four or three receivers.

### 4.2. Reliability Test

This test was performed with the vehicle stopped in the countryside as shown in Figure 7. One by one, each GNSS antenna was partially blocked with a metallic cover during one minute approximately to test sensor reliability.

**Figure 8:**
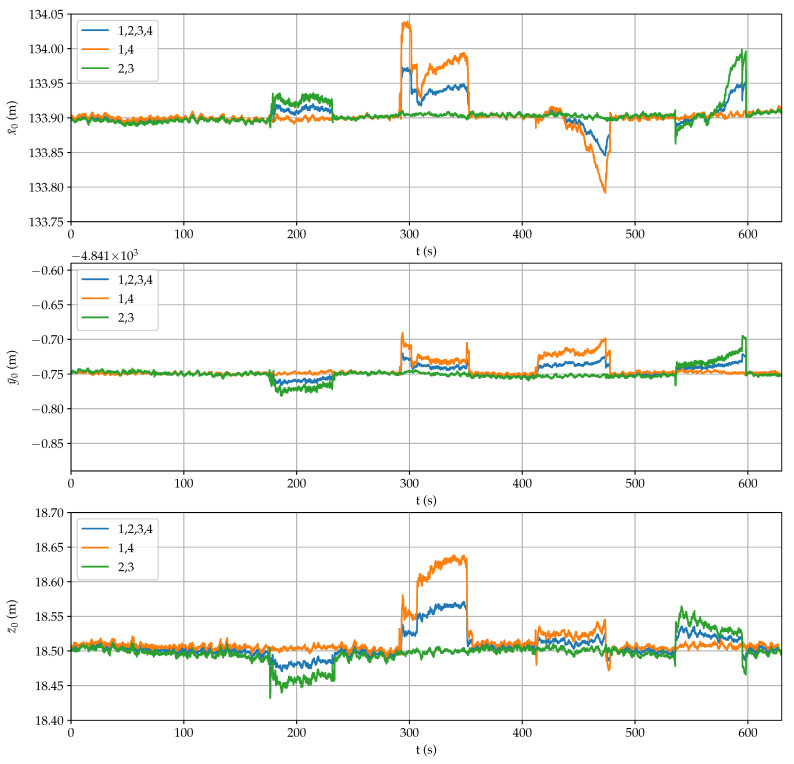
Estimation of position coordinates during the reliability test.

**Figure 9:**
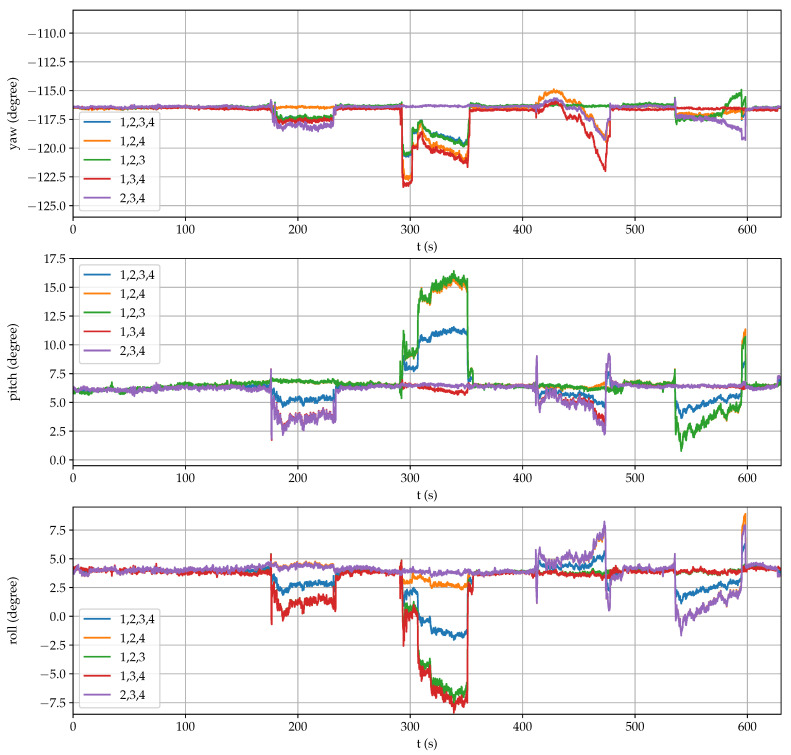
Estimation of posture coordinates during the reliability test.

Figure 8 shows the estimation of NED coordinates when using all (21), the first and the fourth (28) or the second and the third (29) receivers. Similarly, Figure 9 displays the estimation of the three angular coordinates with all the combinations of three and four receivers. In both figures, it can be clearly observed significant estimation changes when an antenna was temporarily blocked.

**Figure 10:**
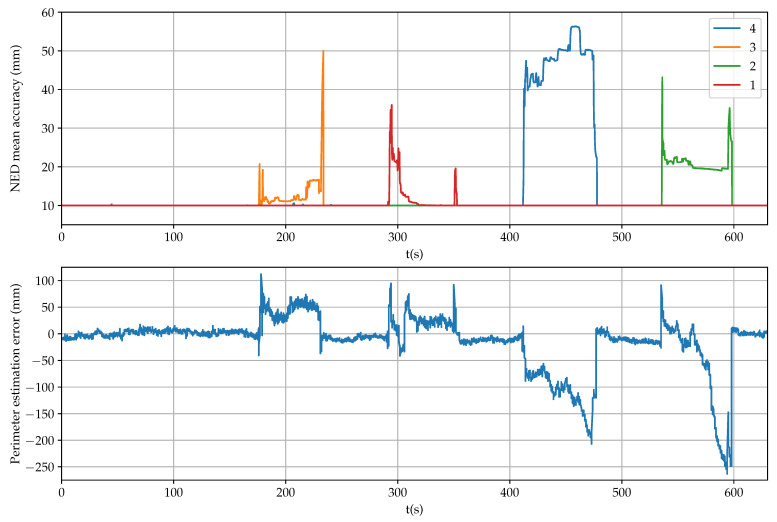
Accuracy estimation during the reliability test.

The mean accuracy provided by each receiver is shown in Figure 10 (up), where it can be observed successive antenna covering, in this order: 3, 1, 4 and 2. Apart from checking the RTK-fixed mode, these values can be employed as a fail indicator for each receiver. However, there is a time period between 325 s to 350 s when the indicator for the first receiver does not detect any error but position and posture estimations were inaccurate.

An additional accuracy indicator is the error in calculating the perimeter of the square from measurements with respect to the data of Table 1 ( 5.389 m). As it is shown in Figure 10 (down), precision degradation can be better detected by using this complementary indicator. Thus, by comparing individually the distances of each vertex with respect to the rest, outlier measurements can be identified adequately when present.

Furthermore, in Figure 8 and Figure 9, it can be observed that the estimations that do not include the failing measurement maintain high accuracy. For example, when the precision of the first receiver degrades in the interval between 290 s to 350 s, good position and posture estimations are provided by the second and third receivers, and by the second, third and fourth receivers, respectively. Therefore, overall accuracy for the sensor system can be maintained by properly detecting a single failure and excluding it from computations.

### 4.3. Dynamic Test

**Figure 11:**
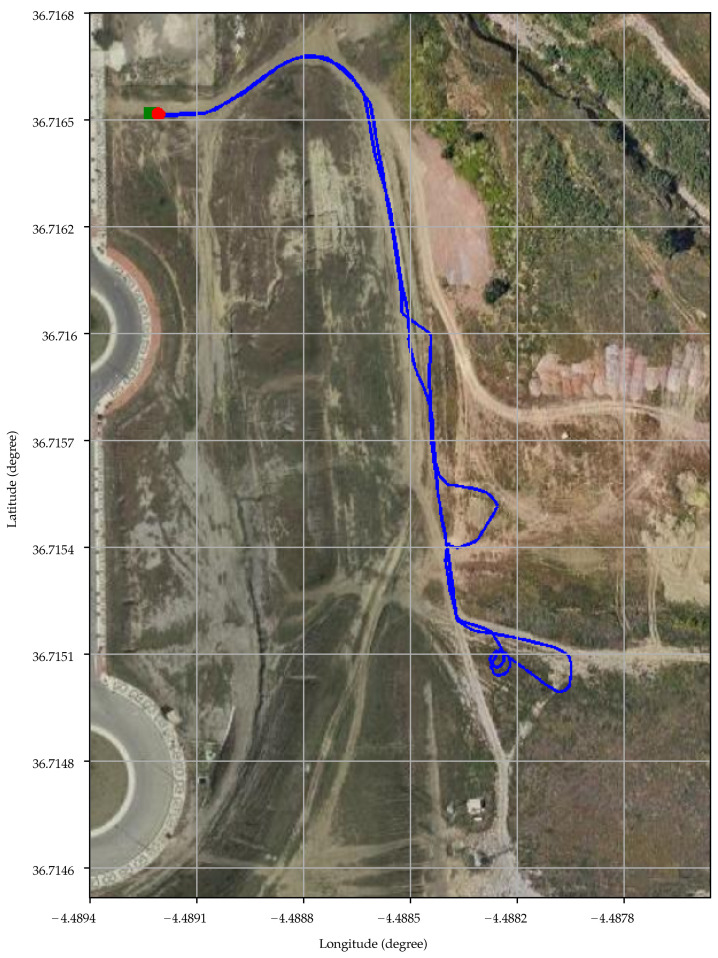
Aerial view of the path followed by the rover on the countryside.

Several experiments were performed by teleoperating the robotic rover on rough countryside. Figure 11 presents an aerial view of one of them using geodetic coordinates for the grid. The beginning and the end of the path, that almost coincide, are marked with a green square and a red circle, respectively. In total, the vehicle travelled 644 m at a mean speed of 4.6 km h^−1^.

**Figure 12:**
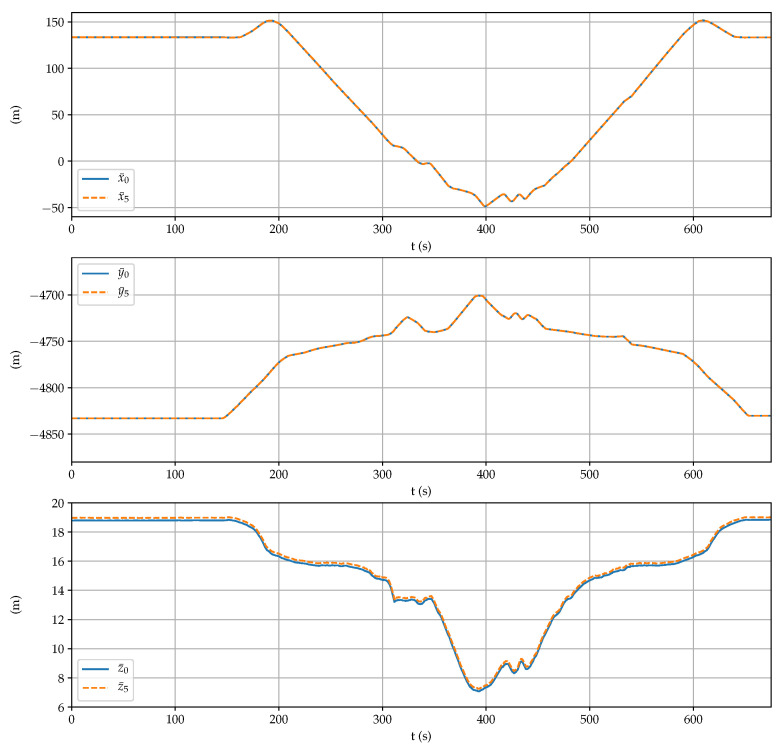
Comparison between the position coordinates obtained by the fifth receiver and the proposed sensor.

Figure 12 shows the three NED coordinates obtained by the proposed sensor system and by the fifth receiver at the center of the square formed by the antennas. There are no appreciable differences between both estimations, with the exception of the step of 0.166 m in the down coordinate (see Table 1). Altogether, the rover went up and, then, under 12 m.

**Figure 13:**
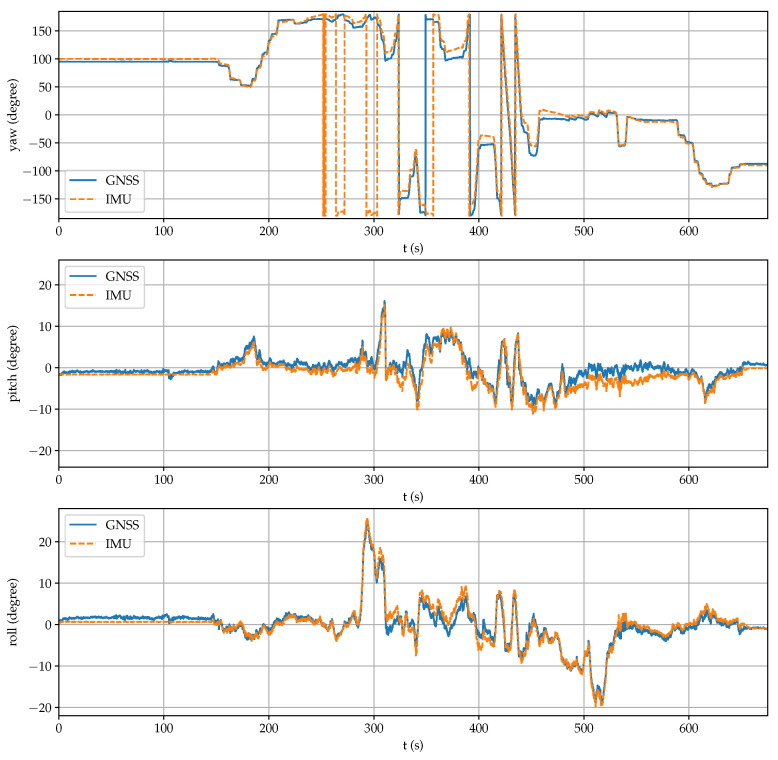
Comparison between the posture coordinates obtained by the IMU and the proposed sensor.

Figure 13 displays the rover posture obtained by the GNSS setup and by the onboard IMU. It can be observed high peaks in the pitch (above 15°) and the roll (above 25°) angles, as well as several complete turns in the yaw angle during the test. Both estimations are very similar, which confirms the good performance of the proposed sensor system.

## 5. Conclusions

A low-cost sensor system composed of four GNSS-RTK receivers connected to a mini computer has been presented in the paper. The placement of three antennas on a vehicle have been analyzed to reduce the uncertainty associated to position and posture estimations with respect to a global reference system. The redundant fourth receiver allows to improve estimations even more and to maintain accuracy when the precision of one of the receivers deteriorates.

Static calibration and reliability tests have been performed with the sensor system mounted on the ground rover Argo XTR. Dynamic experiments on countryside show that this new sensor, in addition to produce reliable positioning, can effectively substitute a high performance IMU to obtain accurate vehicular roll, pitch and yaw angles in real-time.

Future work includes characterizing the achieved pose precision with the robotic rover as well as developing ROS nodes for integrating the proposed sensor system with an IMU for GNSS-denied environments.

## Figures and Tables

**Table 1:** Relative coordinates of the antennas and of the square centroid.

	a (m)	b (m)	c (m)
$v_{1}^{*}$	−0.672	−0.675	−0.004
$v_{2}^{*}$	0.673	−0.676	−0.004
$v_{3}^{*}$	−0.672	0.672	−0.004
$v_{4}^{*}$	0.671	0.678	0.011
$v_{5}^{*}$	−0.004	0.005	0.166
$v_{0}^{*}$ (21)	0	0	0
$v_{0}^{*}$ (28)	0.001	−0.002	−0.004
$v_{0}^{*}$ (29)	0	0.002	0.004

## Abbreviations

The following acronyms are used in the manuscript:

3D	Three-Dimensional
CORS	Continuously Operating Reference Station
ECEF	Earth-Centered, Earth-Fixed
GPS	Global Positioning System
GNSS	Global Navigation Satellite System
IMU	Inertial Measurement Unit
MEMS	Micro Electro Mechanical Systems
NTRIP	Networked Transport of RTCM via Internet Protocol
ROS	Robot Operating System
RTCM	Radio Technical Commission for Maritime Services
SBAS	Satellite Based Augmentation Systems
USB	Universal Serial Bus
